# The Role of Strigolactones in the Regulation of Root System Architecture in Grapevine (*Vitis vinifera* L.) in Response to Root-Restriction Cultivation

**DOI:** 10.3390/ijms22168799

**Published:** 2021-08-16

**Authors:** Yan Xu, Jiyuan Wang, Ruiqi Wang, Lei Wang, Caixi Zhang, Wenping Xu, Shiping Wang, Songtao Jiu

**Affiliations:** Department of Plant Science, School of Agriculture and Biology, Shanghai Jiao Tong University, Shanghai 200240, China; xuyan371372@sjtu.edu.cn (Y.X.); 016150910029@sjtu.edu.cn (J.W.); wangruiqi@sjtu.edu.cn (R.W.); leiwang2016@sjtu.edu.cn (L.W.); acaizh@sjtu.edu.cn (C.Z.); wp-xu@sjtu.edu.cn (W.X.); fruit@sjtu.edu.cn (S.W.)

**Keywords:** grapevine, root restriction, strigolactone, root architecture, gene expression

## Abstract

This study investigated the effects of root-restriction cultivation on the root architecture, endogenous strigolactone (SL) content, and SL-related genes expression in grapevine (*Vitis vinifera* L.). In addition, we clarified the effects of synthetic SL analog GR24 application on grapevine roots to explore the role of SLs in their development. The results showed that the root architecture changed significantly under root-restriction cultivation. At 40 days after transplantation (DAT), the contents of two types of SLs in roots under root restriction were both significantly lower than that in roots of the control. SL content was significantly positively correlated with the expression levels of *VvCCD8* and *VvD27*, indicating that they play vital roles in SLs synthesis. After GR24 treatment for 20 days, the root length was significantly shorter than in the control. A low concentration (0.1 μM) of GR24 significantly reduced the root diameter and increased the fine-root density, while a high concentration (10 μM) of GR24 significantly reduced the lateral root (LR) length and increased the LR density. Concomitantly, GR24 (0.1 μM) reduced endogenous SL content. After GR24 treatment for 5 days, the total content of two tested SLs was highly positively correlated with the expression levels of *VvDAD2*, whereas it was highly negatively correlated with *VvSMAXL4* at 20 days after GR24 treatment. This study helps to clarify the internal mechanism of root-restriction cultivation affecting the changes in grapevine root architecture, as well as further explore the important role of SLs in the growth of grapevine roots in response to root-restriction treatment.

## 1. Introduction

Roots represent one of the most important vegetative organs of plants. It is crucial for plants to absorb water and nutrients from the soil and simultaneously synthesize amino acids, hormones, and other compounds [1]. The growth and development of the root largely determine the absorption efficiency of water and nutrients, which directly affects the photosynthetic products and fruit quality. Grapevine (*Vitis vinifera* L.), a major fruit crop with a released genome, is commercially cultivated worldwide. At present, studies on root growth and development in grapevine have mainly focused on the physiological and applied aspects [2,3]. Only a few of these reports have been focused on the root growth and development of *Vitis*, which further limits its theoretical research [4]. Thus, comprehending the characteristics of root growth and development in grapevine plantlets is vital for its theoretical research, which will contribute to clarifying the regulation of grape quality.

Growing roots present extraordinary plasticity; plants with the same genetic background have different root architecture under different environmental conditions, affecting plant growth and development [5]. In the early 1990s, inspired by potted-plant practices, researchers began to explore cultivation methods that restricted plant roots, i.e., root-restriction (RR) cultivation. Previous studies have shown that grapevines under RR cultivation significantly change their root architecture [6]. The distribution density of the roots increases, along with nutrient and water uptake capacity [7]. The weight of fresh and dry roots, as well as the number and total surface area of roots, decreased with the decrease in rooting-zone volumes, indicating that the rooting-zone volumes significantly affected the root growth and development of grapevine [8]. The root length and diameter of 1 year old grapevine plantlets under the RR cultivation were smaller than those of the control plantlets [9]. Under RR cultivation, the epidermis and cortex thickness of absorbing root in grapevine reached 18.3 μm and 579.1 μm, respectively, much higher than that of the control plantlets, whereas the stele area was 0.004 mm^2^, which was significantly lower than that of the control plantlets. Additionally, grape fruit quality was also significantly improved under RR cultivation [10,11].

Phytohormones, as important plant growth regulators, have a significant impact on the root growth and development process. Strigolactones (SLs), a class of small terpenoid, were acknowledged as a new category of phytohormones, ubiquitous in plants [12]. SLs play an important role in regulating root growth and development and shoot branching, as well as promoting *Orobanche* and *Striga* seed germination [12,13]. GR24, a synthetic analog of SLs, has the highest activity and is the most widely used [14]. Compared with wild-type *Arabidopsis* plants, SL-insensitive and SL-deficient mutants have a shorter primary root and reduced meristem cell numbers [15]. SLs inhibit the formation of adventitious root (AR) in *Arabidopsis* and pea plants by inhibiting the first formative divisions of the founder cell [16]. Rice SL-insensitive or -deficient *dwarf* mutants exhibited a shorter crown root (CR) phenotype [17], and GR24 application complemented the CR defect in SL-deficient rice mutants but not in SL-insensitive rice mutants, indicating that SLs positively regulate the CR length. Previous studies showed that SL biosynthesis and signaling mutants presented increased lateral root (LR) density, while the LR density of tomato plants and *Arabidopsis* decreased after GR24 was externally applied, indicating that SLs can inhibit LR initiation [18,19]. A high concentration (27 μM) of GR24 inhibited root hair (RH) elongation [19], whereas a low concentration (3 μM) of GR24 promoted RH elongation in *Arabidopsis* [18,20]. Several studies applied exogenous GR24 to pea and alfalfa plants and showed that GR24 could promote nodule formation [21,22].

To synthesize SLs, all-*trans*-β-carotens are catalyzed by β-carotenoid isomerase, encoded by *DWARF 27* (*D27*), and two carotenoid cleavage dioxygenases, CCD7 and CCD8, generating carlactone (CL). The CYP711A subfamily of cytochrome P450 oxygenase functions in converting CL into both canonical and noncanonical SLs in vascular plants. In rice, it was demonstrated that CYP711A2/Os900 converts CL into 4-deoxyorobanchol (4DO), and CYP711A3/Os1400 further catalyzes the hydroxylation of 4DO to generate orobanchol (ORO), both of which are endogenous canonical SLs in rice [23]. SLs function in plants through signal transduction mediated by receptor proteins. Rice *Dwarf14* (*D14*) and its homologous gene *DECREASED APICAL DOMINANCE2* (*DAD2*) encode a member of the a/β hydrolase fold family protein, which hydrolyzes the SL molecules into ABC formyl tricyclic lactone (ABC-FTL) and hydroxy methyl butenolide (HMB) [24]. The D14 receptor, along with the F-box protein Dwarf3 (D3) and a repressor of signaling, Dwarf53 (D53), mediates the SL signaling mechanism. In the presence of SLs, the SL receptor forms a complex with D53 and D3, which results in ubiquitination-dependent degradation of D53. Degradation of D53 releases SL signal transduction. On the contrary, in the absence of SLs, D53 suppresses SL signal transduction. Subsequently, SUPPRESSOR OF MAX2 1 (SMAX1)-LIKE6 (SMXL6), SMXL7, and SMXL8 were found to be D53 orthologs which redundantly regulate the SL response in *Arabidopsis* [25,26,27].

The root architecture of grapevine changed significantly after root restriction [28]. SLs play an important role in regulating root growth and development in plants [16,18,19,20]. Thus, we speculated that RR treatment would significantly change grapevine root architecture by affecting the content of SLs or other endogenous hormones. However, the role of SLs in the regulation of grapevine root architecture under RR cultivation still needs to be explored. Therefore, our study firstly investigated the effects of RR cultivation on the grapevine root architecture, endogenous SLs, and their related gene expression levels. Moreover, the effects of GR24 application on grapevine root growth and development, endogenous SL content, and their related gene expression were also clarified, with the aim of establishing the relationship between root architecture and SLs under RR cultivation. This study aimed to clarify the physiological and molecular mechanisms of RR cultivation affecting the changes in root architecture, as well as lay the foundation for further improvements in the theory of the RR cultivation.

## 2. Results

### 2.1. Characterization and Observation of Grapevine Root Development under Root-Restriction and Non-Root-Restriction Cultivation

**Figure 1:**
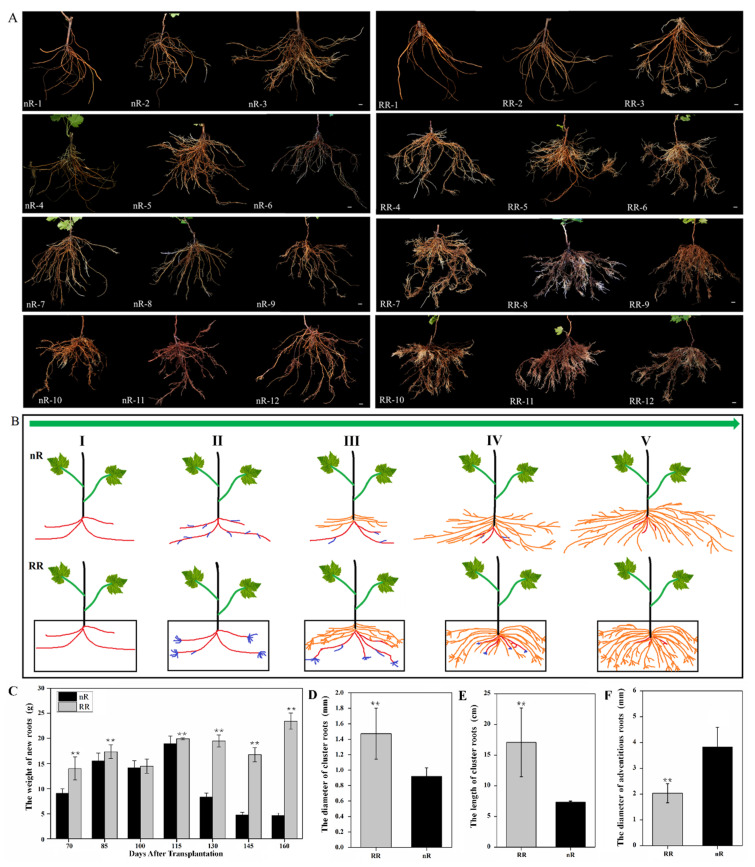
Root parameters and morphological alterations of 1 year old self-rooted grapevine cv. Muscat Hamburg under root-restriction (RR) and non-root-restriction (nR, control) cultivation. The whole-root morphology (**A**) in 12 sampling periods and root model (**B**) of five growth stages are shown; red lines represented old roots from the previous year; blue lines represent new roots growing on old roots; the orange line represents new adventitious roots (ARs). Stages I–V represent the five key stages of root growth in *Vitis vinifera*. Stage I ranges from 0 to 10 days after transplantation (DAT); stage II ranges from 10 to 40 DAT; stage III ranges from 40 to 85 DAT; stage IV ranges from 85 to 115 DAT; stage V ranges from 115 to 205 DAT. The weight of new roots (**C**), the diameter (**D**) and length (**E**) of cluster roots at 130 DAT, and the diameter (**F**) of ARs at 145 DAT are displayed. The data are means ± standard error (SE) of three replicates. Statistical significances were determined using one-way ANOVA, and values (**) are statistically significant from RR and nR according to one-way analysis of variance at a significance level of *p* < 0.01. Scale bar = 1 cm.

**Figure 2:**
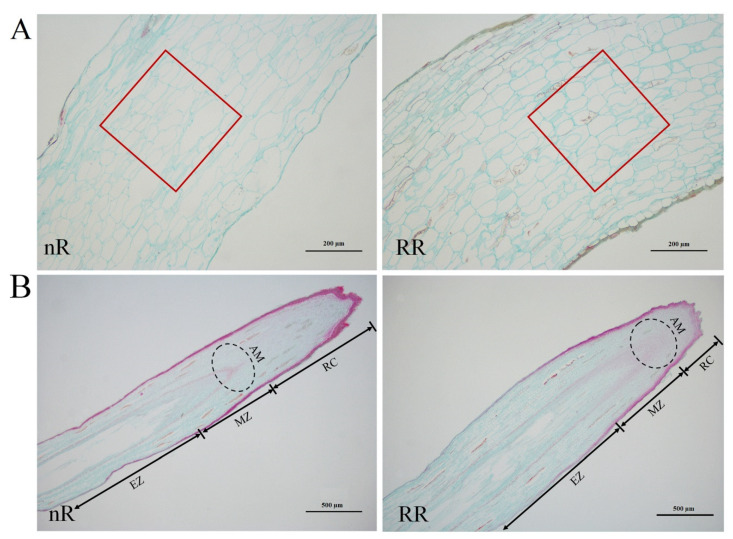
The cell structure of new adventitious roots (ARs) of grapevine cv. ‘Muscat Hamburg’ at 145 days after transplantation (DAT) under root-restriction (RR) and non-root-restriction (nR) cultivation. (**A**) The longitudinal section of new ARs at 2 cm from the root tip; bar = 200 μm. Red boxes are marked to count the number of cells in the same area. (**B**) The longitudinal section of new AR tips; bar = 500 μm. Abbreviations: EZ, elongation zone; MZ, meristem zone; RC, root cap; AM, Apical meristem.

After 10 days of plantlet transplantation, grapevines were sampled and photographed to observe the changes in root phenotypes. Twelve samples were collected during this period. The phenotypes of root sampling at the 12 timepoints under the RR and nR cultivation were recorded as RR1–12 and nR1–12, respectively (Figure 1A). The results showed that, in the first sampling period, new roots under the two cultivation methods were not formed. In the second sampling period, young new roots began to grow in plants in both cultivation methods, but root architecture did not display obvious differences. In the third sampling period, the young roots grew in clusters at the end of ARs under RR cultivation, whereas they were scattered across the entire ARs in the control plants. Starting from the fourth sampling period, the root system architecture began to differ markedly between the RR and control groups, and the differences were obvious at the seventh sampling period (Figure 1A). Under RR cultivation, the ARs disappeared early and promoted a large number of cluster roots in the root cap. The weight of new roots was higher, while the ARs were curved, but thinner in plantlets under the RR group than in those of the control group (Figure 1C,F). Meanwhile, the cluster roots were longer and thicker than in the control group (Figure 1D,E). After RR cultivation, the root architecture of grapevines exhibited changes, which were closely related to changes in the shape and size of the roots. As shown in Figure 2A, the number of cells in new ARs increased, and the cells were arranged tightly under the RR cultivation. Moreover, the longitudinal section of new AR tips showed a shorter root cap (RC) in plantlets under RR than those of the control group (Figure 2B).

### 2.2. Change in Endogenous SL Content of Grapevine Roots under Root-Restriction and Non-Root-Restriction Cultivation

**Figure 3:**
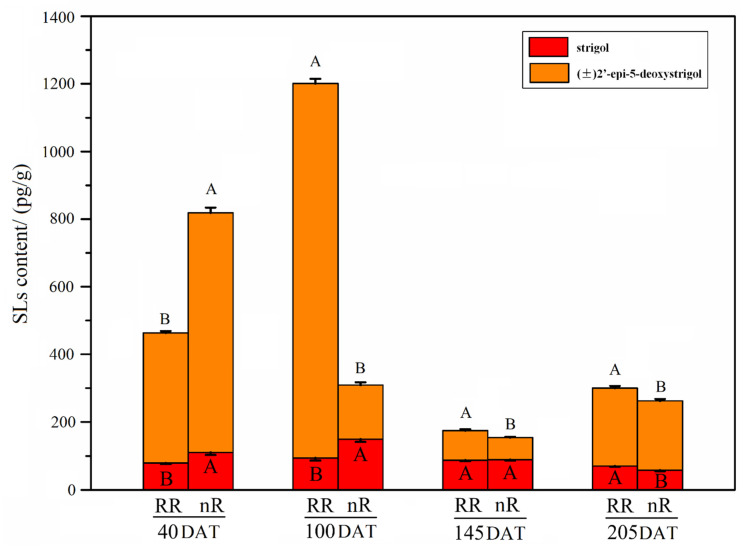
Changes in SL content of grapevine roots at different sampling periods (40, 100, 145, and 205 days after transplantation (DAT)) under root-restriction (RR) and non-root-restriction (nR) cultivation. The data are means ± standard error (SE) of three replicates. Statistical significance was determined using one-way ANOVA; significant differences among means (LSD, *p* < 0.01) are indicated by different capital letters.

To explore the effect of RR on the endogenous SL content during root development, we measured the SL content of young roots from 1 year old self-rooted grapevine plants grown under RR cultivation. The results showed that (±)2′-epi-5-deoxystrigol (DS) and strigol were both detected in the grapevine roots at 40, 100, 145, and 205 days after transplantation (DAT). As shown in Figure 3, the changes in the (±)2′-epi-5-DS content at 40 and 100 DAT were higher than those in strigol. At 40 DAT, the contents of (±)2′-epi-5-DS and strigol in roots of the RR group were both significantly lower than in the control group. Specifically, the total content of both types of SLs in the roots of the RR group was 0.56 times that of the control group at this stage. In contrast, the content of (±)2′-epi-5-DS in the roots of the RR group at 100, 145, and 205 DAT were approximately 6.93, 1.36, and 1.12 times that of the control group, respectively. Specifically, the content of (±)2′-epi-5-DS in the grapevine roots of the RR group at 100 DAT was 1106.9 pg/g, approximately 11.8-fold higher than the strigol content. The content of strigol in the roots of the RR group was significantly higher than that of the control group at 205 DAT, whereas it was significantly lower than that of the control group at 40 and 100 DAT. Overall, the total content of both tested SLs in the grapevine roots of the RR group tended to increase, decrease, and then increase slightly across the four sampling periods, while it showed a trend of first decreasing and then increasing slightly in the control group.

### 2.3. Expression of SL Biosynthesis Genes in Grapevine Roots under Root-Restriction Cultivation

**Figure 4:**
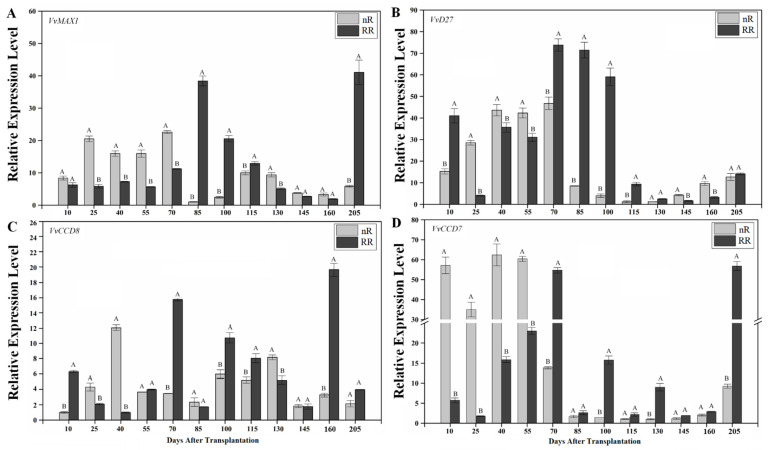
Changes in the expression levels of SL biosynthesis genes in grapevine roots under root-restriction (RR) and non-root-restriction (nR) cultivation. (**A**–**D**) *VvMAX1*, *VvD27*, *VvCCD8*, and *VvCCD7* expression levels in grapevine roots, respectively. The data are means ± standard error (SE) of three replicates. Statistical significance was determined using one-way ANOVA; significant differences among means (LSD, *p* < 0.01) are indicated by different capital letters.

The above results indicated that the endogenous SL content in roots of the RR group changed significantly compared with that of the control group. Thus, we firstly explored the effect of RR on the expression of SL biosynthesis genes (Figure 4). qPCR results showed that RR significantly affected the expression level of four key SL biosynthesis genes, *VvMAX1*, *VvD27*, *VvCCD8*, and *VvCCD7*, at different stages of grapevine root growth. The expression of *VvMAX1* in the RR group at 85 DAT showed the greatest difference, being approximately 38-fold higher than that of the control group (Figure 4A). Subsequently, the difference between RR and control groups gradually decreased until 115 DAT. In the end, the expression of *VvMAX1* in the RR group was significantly higher than that in the control group at 205 DAT, being approximately 6.02 times that of the control group. At 10, 70, 85, 100, and 115 DAT, the expression level of *VvD27* in the RR group was significantly higher than that in the control group (Figure 4B). At 25, 40, and 130 DAT, the expression level of *VvCCD8* in the control group was approximately 2.08, 11.90, and 1.56 times higher than that in the RR group, respectively. However, the expression level of *VvCCD8* in the control group was significantly lower than that in the RR group at 10, 70, 100, 115, and 160 DAT (Figure 4C). The expression of *VvCCD7* was maintained at high levels from 10 to 55 DAT and peaked at 40 DAT in the control group, while it continuously increased until 70 DAT in the RR group (Figure 4D). To connect the SL biosynthesis genes with endogenous SL levels, a correlation between the hormone content and gene expression level was performed in this study (Table S1). Correlation analysis revealed that the expression levels of *VvD27* and *VvCCD8* were significantly positively correlated with the content of (±)2′-epi-5-DS (*r* = 0.960, *p* < 0.01; *r* = 0.811, *p* < 0.05) and the sum of both SLs (*r* = 0.949, *p* < 0.01; *r* = 0.839, *p* < 0.01), indicating that they play vital roles in SL biosynthesis (Table S1). However, correlation analysis showed that the expression levels of the four SL biosynthesis genes were not significantly correlated with the weight of new roots (Table S2).

### 2.4. Expression of SL Signaling Genes in Grapevine Roots under Root-Restriction Cultivation

**Figure 5:**
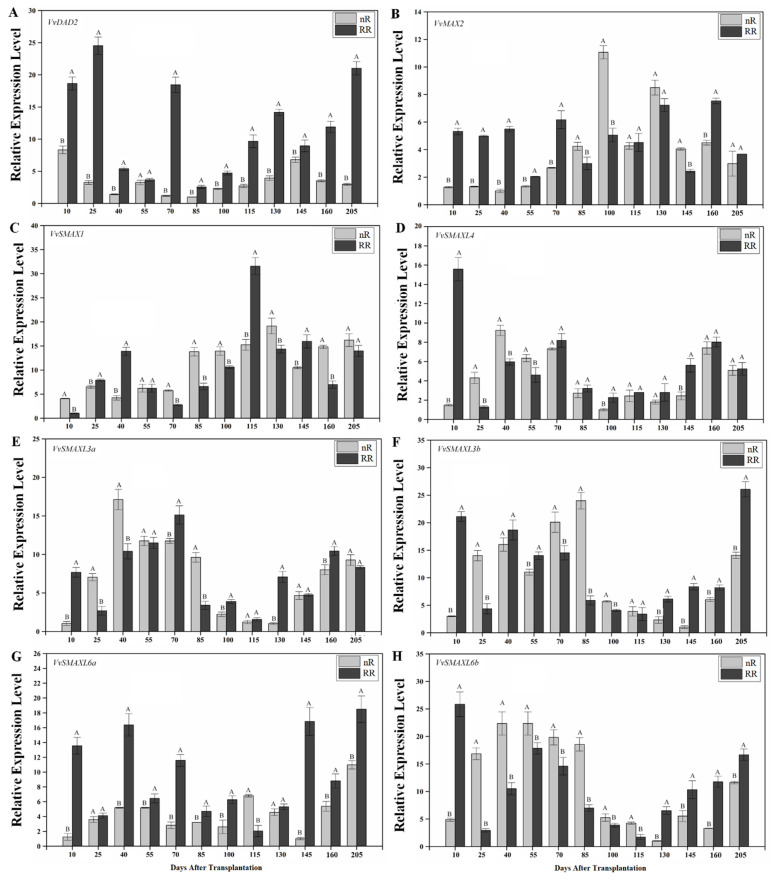
Changes in expression levels of SL signaling genes in grapevine roots under root-restriction (RR) and non-root-restriction (nR) cultivation. (**A**–**H**) *VvMAX2*, *VvDAD2*, *VvSMAX1*, *VvSMAXL4*, *VvSMAXL3a*, *VvSMAXL3b*, *VvSMAXL6a*, and *VvSMAXL6b* expression levels in grapevine roots, respectively. The data are means ± standard error (SE) of three replicates. Statistical significance was determined using one-way ANOVA; significant differences among means (LSD, *p* < 0.01) are indicated by different capital letters.

As shown in Figure 5, RR significantly affected the expression of genes related to SLs signaling in grapevine roots. The expression level of *VvDAD2* after RR cultivation was higher than that in the control group throughout the entire sampling period, and its expression level was highest at 25 DAT in the RR group, being 8.1-fold higher than that in the control group (Figure 5A). As shown in Figure 5B, the data showed that the relative expression of *VvMAX2* in the RR group was significantly higher than that in the nR group during the first five sampling periods, being approximately 4.17, 3.78, 5.45, 1.53, and 2.30 times that in the control group, respectively. In contrast, at 85, 100, 130, and 145 DAT, the expression level of *VvMAX2* in the RR group was significantly lower than that in the control group. As shown in Figure 5C, unlike the expression trends of *VvSMAXL4*, *3a*, *3b*, *6a*, and *6b* genes, the expression of *VvSMAX1* continued to increase from 70 to 115 DAT. The expression level of *VvSMAX1* peaked at 115 and 130 DAT in the RR and control groups, respectively (Figure 5C). The expression trends of *VvSMAXL4*, *VvSMAXL3a*, *VvSMAXL3b*, and *VvSMAXL6b* were similar. These similarities suggest that the above genes play comparable roles in the SL signaling and metabolism pathways. Overall, the expression of these genes tended to increase during the first five sampling phases, then decrease, and finally increase slightly during the last four sampling periods (Figure 5D–F,H). The expression level of *VvSMAXL6a* in the RR group was significantly higher than that in the control group at 10, 40, 55, 70, 85, 100, 145, 160, and 205 DAT, while its expression level was lower than that in the control group at 115 DAT (Figure 5G). Correlation analysis revealed that the expression levels of several SL signaling genes were not significantly correlated with SL content (Table S1) and the weight of new roots (Table S2).

### 2.5. Effect of SL Analog on Grapevine Root System Architecture 

**Figure 6:**
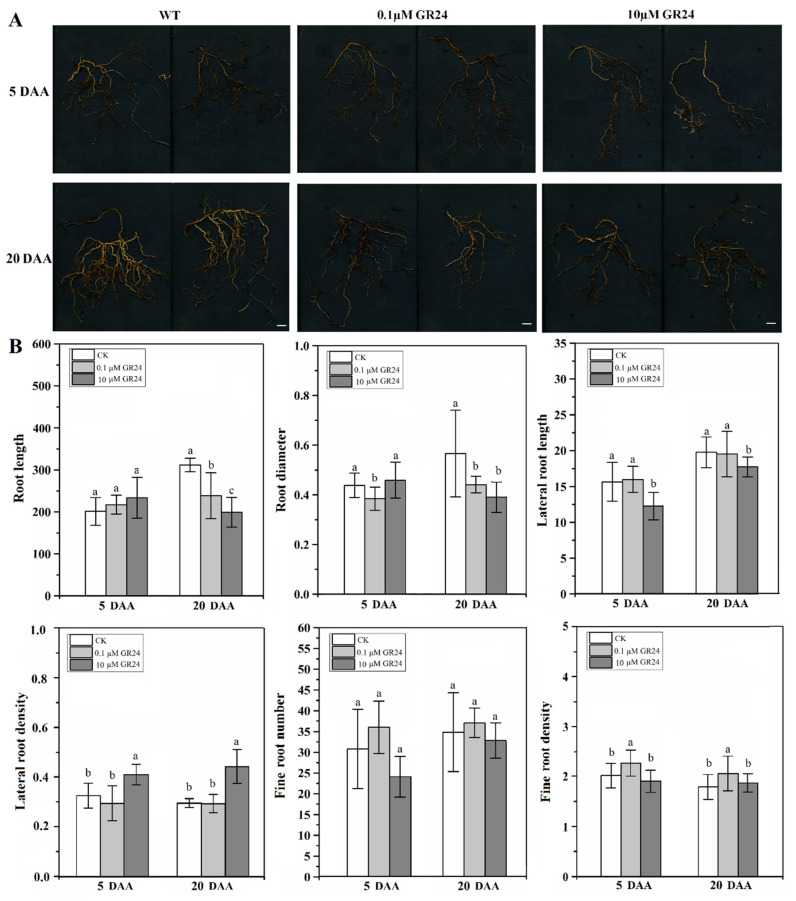
Root morphology (**A**) and parameters (**B**) of grapevine seedlings treated with synthetic SL analog GR24. Bar = 2 cm. The data are means ± standard error (SE, *n* = 12). Statistical significance was determined using one-way ANOVA; significant differences among means (LSD, *p* < 0.05) are indicated by different lowercase letters.

As described above, the root architecture, SL content, and SL-related gene expression were significantly changed after the RR cultivation. Thus, we speculated that SLs might play an important role in manipulating the formation of root architecture under RR cultivation. In this study, to understand the role of SLs in root architecture, the phenotypic characteristics of roots were observed and evaluated at 5 and 20 days after different concentrations of GR24 application (Figure 6A). The analysis of randomly selected ARs showed that changes in root morphological parameters (root length, root diameter, length and density of LRs, and number and density of fine root) were treatment-dependent. As shown in Figure 6B, the root length was not significantly different from that in the control group at 5 days after application (DAA), while 0.1 and 10 μM GR24 treatment led to lengths approximately 0.77 and 0.64 times that in the control group at 20 DAA, respectively. Simultaneously, the root diameter significantly decreased under the 0.1 μM GR24 treatment compared with the control group at 5 and 20 DAA, being approximately 0.88 and 0.78 times that in the control group. On the contrary, the root diameter under 10 μM GR24 treatment was not significantly different from that in the control group on 5 DAA, while it was significantly lower under the 10 μM GR24 treatment than that in the control group at 20 DAA. Thus, the data indicated that SLs are able to inhibit root thickening. By measuring the length and density of LRs on ARs, we found that the inhibition of LR elongation by the 10 μM GR24 treatment occurred at 5 and 20 DAA. LR density in the 10 μM GR24 treatment was significantly higher than that in the control group at 5 and 20 DAA, with the maximums reaching 0.41 and 0.44, respectively. As the number and density of fine roots directly affect the plants in terms of absorption of water and nutrients from soil, we conducted a statistical analysis of the number and density of fine roots after GR24 application. The results showed that the fine-root density in the 0.1 μΜ GR24 treatment was significantly higher than that in the control, being approximately 1.13 and 1.15 times that in the control group at 5 and 20 DAA, respectively, whereas the 10 μΜ GR24 treatment exerted an unobvious effect, implying that low concentrations of GR24 contribute to an increase in the fine-root density. The data indicated that a high concentration (10 μΜ) of GR24 acted as a positive regulator of LR initiation and an inhibitor of LR elongation.

### 2.6. SL Analog Affects Endogenous Hormone Content of Grapevine Root

**Figure 7:**
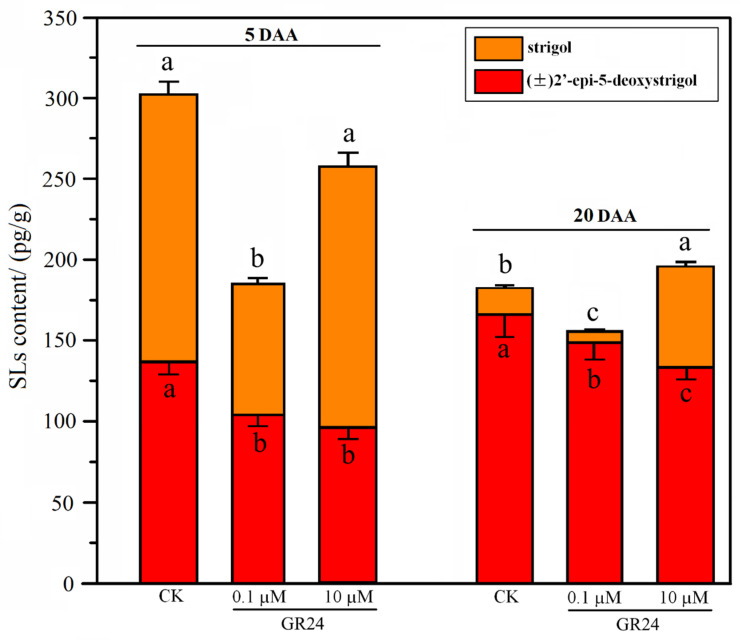
Changes in SL content of grapevine roots treated with synthetic SL analog GR24 at 5 and 20 days after application (DAA). The data are means ± standard error (SE) of three replicates. Statistical significance was determined using one-way ANOVA; significant differences among means (LSD, *p* < 0.05) are indicated by different lowercase letters.

To quantify the difference in growth and development of the grapevine roots treated with the SL analog GR24, two types of SLs, strigol and (±)2′-epi-5-DS, were measured in grapevine roots at 5 and 20 DAA (Figure 7). As shown in Figure 7, GR24 treatment had a significant effect on endogenous SL content in grapevine roots. At 5 DAA, GR24 treatment resulted in a significant decrease in the total content of both types of SLs in grapevine roots. At 5 and 20 DAA, the content of (±)2′-epi-5-DS in the grapevine roots under the 0.1 and 10 μM GR24 treatments was lower than that in the control group. At 5 DAA, the content of strigol significantly decreased under the 0.1 GR24 treatment, whereas it was not significantly different from that in the control group under the 10 μM GR24 treatment. At 20 DAA, the content of endogenous strigol was 51.11 pg/g in the grapevine roots under 10 μM GR24 treatment, being approximately 3.68 times that in the control group. The results indicated that 10 μM GR24 significantly increased the content of endogenous strigol, whereas 0.1 μΜ GR24 treatment showed the opposite effect at 20 DAA. Additionally, the data showed that the hormone level of strigol was higher in the early stage after GR24 treatment. To combine the endogenous SL levels with root morphological parameters after GR24 treatment, their correlation analysis was performed in this study. Correlation analysis showed that the endogenous SL levels were not significantly correlated with root morphological parameters in *V. vinifera* at 5 DAA (Table S3) and 20 DAA (Table S4).

### 2.7. GR24 Treatment Affects the Expression of SL Biosynthesis Genes

**Figure 8:**
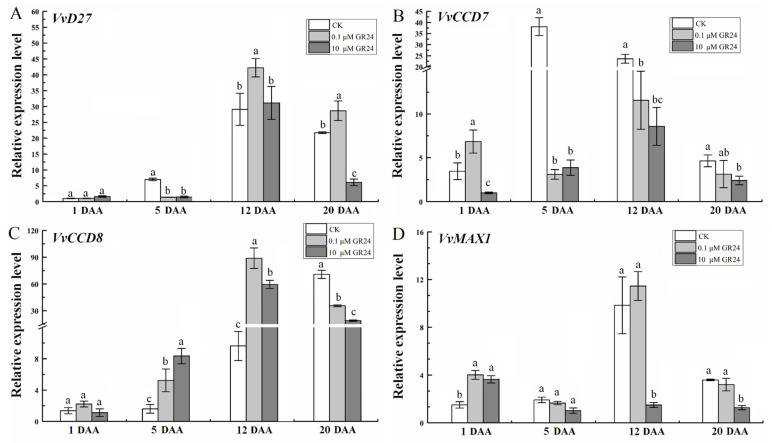
Changes in the expression levels of SL biosynthesis genes in grapevine roots treated with GR24 at 1, 5, 10, and 20 days after application (DAA). (**A**–**D**) *VvD27*, *VvCCD7*, *VvCCD8*, and *VvMAX1* expression levels in grapevine roots treated with GR24, respectively. The data are means ± standard error (SE) of three replicates. Statistical significance was determined using one-way ANOVA; significant differences among means (LSD, *p* < 0.05) are indicated by different lowercase letters.

As shown in Figure 8, exogenous GR24 treatment had different effects on the expression of SL biosynthesis genes in grapevine roots. There were certain similarities in the expression patterns of the genes; the expression levels of *VvD27*, *VvCCD8*, and *VvMAX1* were low at 1 and 5 DAA, but increased significantly at 12 DAA (Figure 8A). As shown in Figure 8B, the expression level of *VvCCD7* in the grapevine roots treated with GR24 was inhibited compared with the control group at 5, 12, and 20 DAA, while there was no significant difference in the expression level after treatments with different concentrations of GR24. At 20 DAA, the expression of *VvCCD7* was significantly positively correlated with root length and diameter, with correlation coefficients of 1 and 0.999, respectively (Table S5). As shown in Figure 8C, compared with the control, GR24 treatment significantly inhibited the expression of *VvCCD8* at 20 DAA, being approximately 0.50 and 0.27 times that in the control group, respectively. Interestingly, the expression of *VvCCD8* was also significantly positively correlated with root length and diameter at 20 DAA, with correlation coefficients as high as 0.999 (Table S5). As shown in Figure 8D, GR24 treatment significantly induced the upregulation of *VvMAX1* expression at 1 DAA. At 12 and 20 DAA, the expression level of *VvMAX1* under 0.1 μM GR24 treatment was not significantly different from that in the control group, while the 10 μM GR24 treatment significantly inhibited the expression of *VvMAX1*. Interestingly, the expression of *VvMAX1* was significantly positively correlated with the length of LRs at 20 DAA, with a correlation coefficient of 0.999 (Table S5).

### 2.8. GR24 Treatment Affects the Expression of SL Signaling-Related Genes

**Figure 9:**
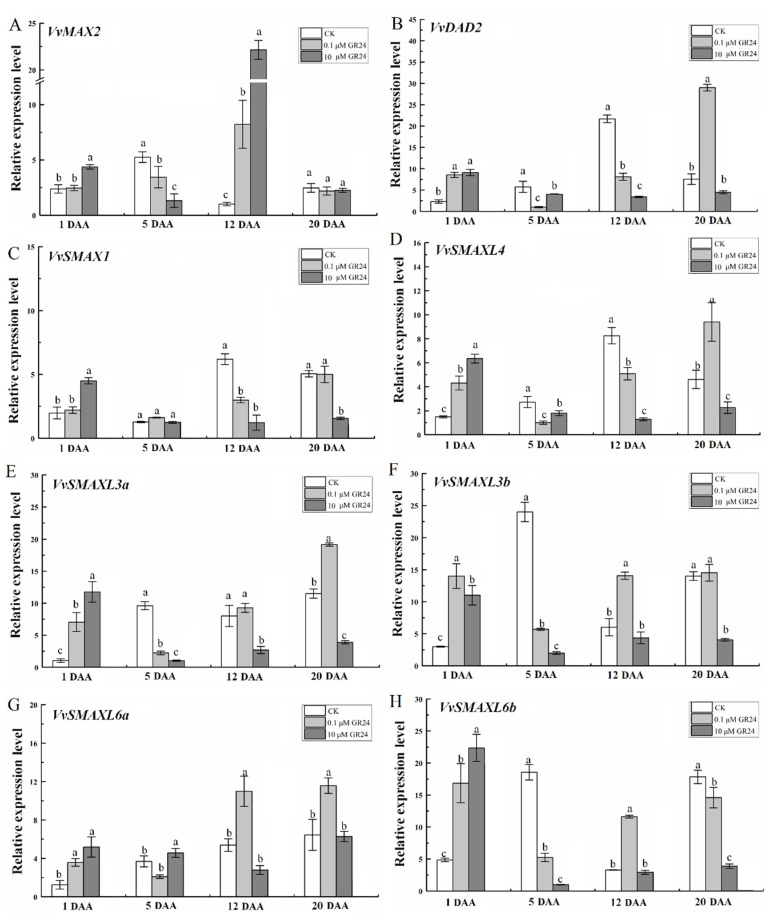
Changes in the expression levels of SL signaling genes in grapevine roots treated with GR24 at 1, 5, 10, and 20 days after application (DAA). (**A**–**H**)*VvMAX2*, *VvDAD2*, *VvSMAX1*, *VvSMAXL4*, *VvSMAXL3a*, *VvSMAXL3b*, *VvSMAXL6a*, and *VvSMAXL6b* expression levels in grapevine roots treated with GR24, respectively. The data are means ± standard error (SE) of three replicates. Statistical significance was determined using one-way ANOVA; significant differences among means (LSD, *p* < 0.05) are indicated by different lowercase letters.

To further clarify how exogenous GR24 treatment affects the expression levels of SL signaling-related genes in grapevine roots, we analyzed the expression patterns of eight genes, namely, *VvMAX2*, *VvDAD2*, *VvSMAX1, VvSMAXL4,* and *VvSMAXL3a, 3b, 6a*, and *6b*, in this study. As shown in Figure 9, GR24 treatment also had a high impact on the expression of SL signaling-related genes. As shown in Figure 9A, the expression of *VvMAX2* was significantly lower than that in the control group at 5 DAA, and its expression was positively correlated with the change in root length, with a correlation coefficient of 0.999 (Table S6). As shown in Figure 9B, at 5 and 12 DAA, the two treatments of 0.1 and 10 μM GR24 significantly inhibited the expression of *VvDAD2*. As a receptor of SLs, the expression of *VvDAD2* was significantly positively correlated with the total content of both SLs at 5 DAA, with a correlation coefficient of 1 (Table S6). In addition, the expression levels of *VvSMAXL3a*, *3b*, and *6b* were significantly positively correlated with the strigol content, with a correlation coefficient of 0.998–0.999 (Table S6). However, the expression level of *VvSMAXL4* was significantly negatively correlated with the content of both SLs at 20 DAA, with a correlation coefficient of 0.997 (Table S5). LR density was significantly positively correlated with *VvSMAXL6a* at 5 DAA (Table S6) and significantly negatively correlated with *VvSMAX1* and *VvSMAXL3b* at 20 DAA (Table S5).

## 3. Discussion

### 3.1. Effects of Root Restriction on Grapevine Root Architecture

The shape of the root system is affected by external environmental factors, which, in turn, affect the absorption and utilization efficiency of water and nutrients by plants [29]. The absorption of nutrients and water by roots depends on phenotypic parameters such as number, length, and effective absorption area of new roots [30,31,32]. Therefore, the environmental conditions of root growth are essential for regulating the vegetative and reproductive growth of aboveground and underground parts of plants. Previous studies indicated that the root growth of grapevine contains two peak periods, which are from early May to mid-June and from August to mid-September [33,34]. In our study, growth speed during the first root-growth peak period was significantly higher than that during the second peak period (Figure 1). These results show that the roots of 1 year old self-rooted grapevines occurred in large numbers from the third (7 May 2019) to the sixth sampling observation (22 June 2019). The new clustered roots appeared on the old ARs at 40 DAT. Subsequently, new ARs appeared at 85 DAT. From the beginning of the seventh to the tenth sampling period, some new roots appeared and reached a stable state.

Root-zone regulation can increase root vitality and density in apple trees [35]. Under RR cultivation, fibrous root of *Osmanthus fragrans* sprouted considerably, fine root increased, and plantlets formed root masses earlier and faster than under traditional cultivation. Under RR cultivation, the length, volume, and surface area of roots increased by 62.37%, 32.35%, and 26.23%, respectively, compared with the control group [36]. On the basis of the annual observation of the 1 year old ‘Muscat Hamburg’ grapevine root architecture, we summarize the characteristics of grapevine root formation. Compared with the control group, ARs and LRs occurred in large numbers, and ARs were curved, but thinner in plantlets after RR cultivation, which is consistent with previous studies [37]. In addition, our findings showed that the new roots of grapevine under RR cultivation did not stop growing; however, growth stopped in the control group at later time points of the growth phase (Figure 1C), which may be significantly related to the change in external environment caused by the difference in rooting-zone volume of grapevine between the RR and control groups. Previous studies have also shown that the growth and development of the root system present complex behavioral patterns owing to changes in the external environment, similar to certain characteristics of animals that also occur with changes in the environment conditions [38]. Thus, we speculated that the grapevines needed more water and nutrients to grow alongside trees; however, because of the limitation of rooting-zone volume, they had to increase the number of new roots to absorb more water and nutrients.

### 3.2. Effect of Root Restriction on Hormone Content and SL-Related Gene Expression in Grapevine Roots

Roots represent an important organ for synthesizing endogenous hormones, such as SLs, cytokinin (CTK), and abscisic acid (ABA). Previous studies indicated that RR changed the content of endogenous hormones, such as ABA, in the roots, leaves, sap, and fruits of grapevines [39,40]. Some reports revealed that container size is closely correlated with CTK content [41]. Similarly, RR treatment reduces auxin levels in grape berries but increases brassinosteroid (BR) content [42]. The biological function of SLs in plants is inseparable from the role of auxins [18]. Under RR cultivation, the expression of *VvDAD2* and *VvMAX2* was significantly upregulated in this study, which is consistent with the upregulation of the auxin biosynthesis genes, *VvTAA1*, *VvTAR2*, *VvYUC2*, and *VvYUC4*, during some sampling periods [43]. Our results showed that RR has varying degrees of impact on the expression levels of SL biosynthesis- and signaling-related genes, as well as SL content in grapevine roots. Correlation analysis showed that the expression levels of *VvD27* and *VvCCD8* were significantly positively correlated with the SL content in grapevine roots, indicating that they play vital roles in SL synthesis (Table S1). Moreover, the expression levels of *VvCCD8* were significantly positively correlated with the length and diameter of grapevine roots at 20 DAA (Table S5). Thus, the above results indicated that *VvCCD8* manipulates the biosynthesis of endogenous SLs, thereby regulating root growth and development in grapevines.

### 3.3. Effect of SLs on the Growth and Development of Grapevine Roots

As a new phytohormone, SLs play an important role in regulating the root growth and development process in plants [15,16,18]. GR24 is the most extensively applied synthetic SL, with analogous bioactivity to that of endogenous SLs [12]. A previous study indicated that the length, surface area, diameter, and volume of roots in grapevine ‘Cabernet Sauvignon’ plantlets sprayed with GR24 significantly increased under drought stress [44]. In our study, a remarkable decrease in root length and diameter occurred when plantlets were treated with 0.1 and 10 μM GR24, in comparison with the control group at 20 DAT, which is inconsistent with the results of Li et al. [44]. The differences may be due to the fact that the changes in root architecture reported by Li et al. [44] occurred under drought stress. Thus, we speculate that the effect of drought stress signals on root growth and development may be much greater than that of GR24 hormone signals. Moreover, treatment with GR24 at low concentrations (1.8 × 10^−4^ to 1.8 μM) was observed to accelerate LR development, whereas high concentrations (18 μM) demonstrated an inhibitory effect in rapeseed [45]. Our results showed that a high concentration (10 μM) of GR24 inhibited LR length, in comparison with control plantlets. However, treatment with a low concentration (0.1 μM) of GR24 did not significantly affect the LR length of grapevine in this study, which is not consistent with the findings for rapeseed [45]. This may be attributed to the difference in sensitivity between grapevine and rapeseed, belonging to different plant families, to SL hormone concentrations. Additionally, our data indicated that low and high concentration of GR24 both inhibited root elongation, which is inconsistent with the findings of Koltai et al. [46,47] in *S. lycopersicum*. This may be due to the fact that grapevine and tomato belong to different plant families, and there are many genetic, physiological, and developmental differences between the two plant species. In higher plants, the initiation and elongation of LRs and ARs are vital for the formation of root architecture. The data showed that GR24 inhibited the elongation and thickening of root, as well as LR elongation, and it improved the density of LRs and fine root (Figure 6). Meanwhile, the total content of two types of SLs in roots in the RR group was higher than that in the control group at 100, 145, and 205 DAT (Figure 3). Combining the results of the current study with previous reports [19,35,37,48,49], we speculate that SLs are involved in the regulation of root architecture mainly via affecting the initiation and thickening of ARs, as well as the number of LRs. It is worth mentioning that the mechanism underlying the SL-mediated regulation of root architecture and the interaction network of SLs and other phytohormones under RR cultivation warrant further research.

## 4. Materials and Methods

### 4.1. Plant Materials

The experiment was conducted in 2019 in a greenhouse (65–85% relative air humidity) located at Shanghai Jiao Tong University in Shanghai, China (31°11 N, 121°29 W). We selected 150 1-year old self-rooted grapevine cv. ‘Muscat Hamburg’ under RR and traditional ground planting (as the control group). For the RR group, grapevines were planted at 40 cm depth with 30 cm diameter ridges from plastic trays to isolate the root zone from outside the ground. The cultivation medium was a mixture of loam, manure, and river sand (1:1:1; *v*:*v*:*v*). The grapevine plantlets of the control group were planted using the same medium in the field with unrestricted rooting zones. All vines were placed at a spacing of 60 × 60 cm in north–south-oriented rows and trained using a single tendril growth system. Fertilizer and water management was performed according to the method described by Wang et al. [11].

The experimental plantlets for GR24 treatment were also 1 year old self-rooted grapevine cv. ‘Muscat Hamburg’ with the same growth conditions. They were planted in 25 cm diameter plastic pots filled with perlite to fix the plants. Before transplantation, the grapevine roots were cleaned and then soaked in distilled water for 1–2 days to improve plantlet survival rate. An automatic irrigation system was used to supply Hoagland nutrient solution to each plantlet individually.

### 4.2. Plant Sampling

The experimental materials were transplanted on 29 March 2019. Root-restriction (RR) and non-root-restriction (nR, control) plants were observed and sampled at 10, 25, 40, 55, 70, 85, 100, 115, 130, 145, 160, and 205 DAT. At each sampling time point, six vines were selected for sampling from the RR and control groups, respectively, and each plantlet was used for sampling only once. To ensure the integrity of the root system, the soil around the root was washed with running water. Root digging range was centered on the trunk of the grapevine, and the crown width was the reference standard. When sampling the RR plantlets, the soil in the ridges was wetted with running water, and the ridges were disassembled. The soil was also washed away with running water, and the whole-root system was removed, cleaned, and dried with absorbent paper. The grapevine whole-root system was evenly spread out and photographed on a black background. Simultaneously, the young new roots were collected, weighed, and immediately placed in liquid nitrogen, before storing at −80 °C.

### 4.3. Exogenous GR24 Treatment

After 40 DAT, the new shoots and roots of the grapevines grew rapidly, and the plantlets with consistent growth were selected for subsequent GR24 treatment experiments. Each plantlet was irrigated evenly with 450 mL of GR24 solution on the basis of previous observations. We set three GR24 concentration gradients: 0 μM (control), 0.1 μM, and 10 μM, following previous reports [45,50] and prepared a total of 24 plantlets for each condition. GR24 was purchased from Beijing Daqin Science Co., Ltd. To ensure effectiveness, the above treatments were performed three times with an interval of 6 days each. Young roots were collected at 1, 5, 12 and 20 days after the third treatment application, immediately placed in liquid nitrogen, and stored at −80 °C. Three biological replicates were used for each treatment at different sampling timepoints.

### 4.4. Root Morphological Evaluation

At each timepoint, three grapevines were selected from each treatment group, and the perlite around the root was carefully washed to avoid damaging the root system during sampling. The root system was carefully placed in a rectangular tray with water and evenly distributed with tweezers to reduce root overlap. Subsequently, four ARs were randomly selected from each plantlet for scanning with an Epson Perfection V700 Photo Scanner (Epson, Nagano, Japan). Lastly, the WinRHIZO root analysis system (Regent Instruments, Quebec, Canada) was used to analyze the root morphology parameters, including root length, diameter, surface area, and tip number.

### 4.5. Paraffin Section Sample Preparation

After 145 DAT, the root systems from RR and control groups were sampled for cellular structure analysis. The observed root samples included a longitudinal section about 2 cm from the top of the young root tip and a longitudinal section through a young root tip. The preparation of paraffin sections was based on the method described by An [51]. The slides were analyzed and photographed using an BX61 microscope (Olympus, Tokyo, Japan).

### 4.6. SL Content Measurement

All tests were performed using an ultra-performance liquid chromatography (UPLC) system (Waters, Milford, MA, USA) combined with a 5500 QTRAP^®^ mass spectrometer (MS) system equipped with an electrospray ionization source (AB SCIEX, Foster City, CA, USA). Data acquisition and processing were performed using Analyst 1.6.2 software (AB SCIEX, Foster City, CA, USA). Plant hormones were isolated using a BEH C18 column (130 Å, 1.7 μm, 2.1 × 100 μm). (±)2′-epi-5-DS and strigol were quantified as described by Rial et al. [52]. Standard substances of (±)2′-epi-5-DS and strigol were purchased from OlChemIm (Olomouc, Czech Republic). Final SL content was expressed as pg/g fresh weight (FW).

### 4.7. RNA Isolation and cDNA Synthesis

Total RNA was extracted from grapevine samples using the cetyltrimethylammonium bromide (CTAB) method [53,54]. The concentration of total RNA was evaluated using NanoDrop (Thermo Fisher Scientific Inc., Waltham, MA, USA); the OD260/280 ratio was close to 2.0, while the OD260/230 ratio was >2.0. Subsequently, RNA integrity was assessed using ethidium bromide staining and denatured agarose gel electrophoresis [55,56]. RNA samples were digested with DNase I (TaKaRa, Shiga, Japan) to avoid genomic DNA contamination. RNA samples (1.0 mg) were used for the construction of cDNA libraries using the PrimeScript TM First Strand cDNA synthesis kit (TaKaRa, Ishiyama, Japan), following the manufacturer’s protocol.

### 4.8. Quantitative Real-Time PCR (qRT-PCR) Analysis

The primers used for amplifying the SL-related genes were designed using the Primer-BLAST online program (https://www.ncbi.nlm.nih.gov/tools/primer-blast/index.cgi?LINK_LOC=BlastHome; accessed on 27 February 2020). The qRT-PCR assay was performed using an iCycler iQ5™ Real Time PCR Detection System (Bio-Rad, Hercules, CA, USA). The PCR mixture (20 µL) included 10 µL of 2× TB Green II mix, 2 µL of cDNA template, 0.4 µL each of 10 µM forward primer and 10 µM reverse primer, and RNase-free water (7.2 µL). The operational parameters of the qRT-PCR assay were determined as described by Jiu et al. [57]. Each sample was analyzed in three technical replicates, and the 2^−ΔΔCT^ method was used to compute the relative expression level of each tested gene [58]. *KyActin1* was examined in parallel as an internal reference control, to standardize the gene expression levels [59]. The lowest expression levels of the samples were manually set to 1. The primers used for qRT-PCR are listed in Table S7.

### 4.9. Statistical Analysis

The experiment was arranged in a completely randomized design with three replications. The data were statistically analyzed using SAS software (Version 9.2, SAS Institute Inc., Cary, NC, USA). Data were represented as means ± standard error (SE) of three biological replicates. Statistical differences were determined using one-way ANOVA at a significance level of *p* < 0.05 or *p* < 0.01. The correlation between two variables was verified using Pearson’s correlation coefficient.

## 5. Conclusions

This study showed that the root architecture and cell morphology of grapevine roots changed significantly under RR cultivation. At 40 DAT, the contents of two types of SLs in roots under root restriction were both significantly lower than that in roots of the control. At 20 DAA, the root length was significantly shorter than in the control group. A low concentration of GR24 significantly reduced the root diameter and increased the fine-root density, while a high concentration of GR24 significantly reduced the LR length and increased the LR density. In addition, 0.1 μM GR24 treatment significantly reduced endogenous SL content. At 5 DAA, the total content of two tested SLs was highly positively correlated with the expression levels of *VvDAD2*, whereas it was highly negatively correlated with *VvSMAXL4* at 20 DAA. After GR24 treatment for 20 days, the expression levels of *VvCCD7* and *VvCCD8* were significantly positively correlated with the root length and diameter, and VvMAX1 was significantly positively correlated with LR length, while *VvSMAX1* and *VvSMAXL3b* were both significantly negatively correlated with LR density, indicating that these genes responded to GR24 treatment and, thus, regulated the grapevine root architecture. Hence, this study helps to clarify the internal mechanism of RR cultivation affecting the changes in grapevine root architecture.

## Data Availability

Not applicable.

## Supplementary Materials

The following are available online at https://www.mdpi.com/article/10.3390/ijms22168799/s1.
