# Supplementary material for: The Role of Strigolactones in the Regulation of Root System Architecture in Grapevine (Vitis vinifera L.) in Response to Root-Restriction Cultivation
Source: Int J Mol Sci. 2021 Aug 16;22(16):8799. doi: 10.3390/ijms22168799 (PMC8395845; doi:10.3390/ijms22168799)
Supplement: Supplementary file 1 [file ijms-22-08799-s001.zip › Table S1.pdf]

Table S1. Correlation between gene expression levels and endogenous SLs content in *V. vinifera* roots.

Code	Annotation	(±)-2'-epi-5-deoxystrigol	strigol	(±)-2'-epi-5-deoxystrigol and strigol
1	*VvMAX1*	0.688	-0.139	0.67
2	*VvCCD7*	0.357	-0.109	0.346
3	*VvCCD8*	0.811*	0.465	0.839**
4	*VvD27*	0.960**	-0.026	0.949**
5	*VvDAD2*	-0.275	-0.427	-0.305
6	*VvMAX2*	-0.137	0.646	-0.087
7	*VvSMAX1*	-0.567	-0.34	-0.588
8	*VvSMAXL4*	0.123	-0.337	0.096
9	*VvSMAXL3a*	0.245	-0.243	0.224
10	*VvSMAXL3b*	-0.048	-0.432	-0.08
11	*VvSMAXL6a*	-0.188	-0.612	-0.233
12	*VvSMAXL6b*	0.048	-0.207	0.032

"*" and "**" after the correlation coefficients represent the correlation between their mRNA expression levels and endogenous hormone levels significantly different at $P < 0.05$ and $P < 0.01$, respectively.
