# Supplementary material for: The Role of Strigolactones in the Regulation of Root System Architecture in Grapevine (Vitis vinifera L.) in Response to Root-Restriction Cultivation
Source: Int J Mol Sci. 2021 Aug 16;22(16):8799. doi: 10.3390/ijms22168799 (PMC8395845; doi:10.3390/ijms22168799)
Supplement: Supplementary file 1 [file ijms-22-08799-s001.zip › Table S2.pdf]

Table S2. Correlation between SLs-related gene expression levels and the weight of new roots in *V. vinifera*

Code	Annotation	The weight of new roots
1	*VvMAX1*	-0.009
2	*VvCCD7*	-0.023
3	*VvCCD8*	0.400
4	*VvD27*	-0.043
5	*VvDAD2*	0.360
6	*VvMAX2*	0.104
7	*VvSMAX1*	0.125
8	*VvSMAXL4*	-0.032
9	*VvSMAXL3a*	-0.048
10	*VvSMAXL3b*	0.033
11	*VvSMAXL6a*	0.319
12	*VvSMAXL6b*	0.084
