# Supplementary material for: The Role of Strigolactones in the Regulation of Root System Architecture in Grapevine (Vitis vinifera L.) in Response to Root-Restriction Cultivation
Source: Int J Mol Sci. 2021 Aug 16;22(16):8799. doi: 10.3390/ijms22168799 (PMC8395845; doi:10.3390/ijms22168799)
Supplement: Supplementary file 1 [file ijms-22-08799-s001.zip › Table S3.pdf]

**Table S3. Correlation between root parameters and SLs content in *V. vinifera* roots at 5 DAA.**

| Code | Root phenotype | (±)-2'-epi-5-deoxystrigol | strigol | (±)-2'-epi-5-deoxystrigol and strigol |
|---|---|---|---|---|
| 1 | Root length | -0.038 | -0.946 | -0.377 |
| 2 | Root diameter | 0.951 | 0.056 | 0.789 |
| 3 | Lateral root length | -0.533 | 0.597 | -0.212 |
| 4 | Lateral root density | 0.672 | -0.448 | 0.379 |
| 5 | Fine root number | -0.802 | 0.267 | -0.55 |
| 6 | Fine root density | -0.944 | -0.032 | -0.774 |
