# Supplementary material for: The Role of Strigolactones in the Regulation of Root System Architecture in Grapevine (Vitis vinifera L.) in Response to Root-Restriction Cultivation
Source: Int J Mol Sci. 2021 Aug 16;22(16):8799. doi: 10.3390/ijms22168799 (PMC8395845; doi:10.3390/ijms22168799)
Supplement: Supplementary file 1 [file ijms-22-08799-s001.zip › Table S4.pdf]

Table S4. Correlation between root parameters and SLs content in *V. vinifera* roots at 20 DAA.

Code	Root phenotype	(±)-2'-epi-5-deoxystrigol	strigol	(±)-2'-epi-5-deoxystrigol and strigol
1	Root length	-0.655	0.991	-0.159
2	Root diameter	-0.602	0.98	-0.09
3	Lateral root length	-0.963	0.901	-0.675
4	Lateral root density	0.989	-0.841	0.761
5	Fine root number	-0.923	0.438	-0.987
6	Fine root density	-0.383	-0.324	-0.812
