# Supplementary material for: The Role of Strigolactones in the Regulation of Root System Architecture in Grapevine (Vitis vinifera L.) in Response to Root-Restriction Cultivation
Source: Int J Mol Sci. 2021 Aug 16;22(16):8799. doi: 10.3390/ijms22168799 (PMC8395845; doi:10.3390/ijms22168799)
Supplement: Supplementary file 1 [file ijms-22-08799-s001.zip › Table S5.pdf]

Table S5. Correlation between SLs-related gene expression levels and the parameters and SLs content of grapevine roots at 20 DAA.

Code	Annotation	(±)-2'-epi-5-deoxystrigol	strigol	(±)-2'-epi-5-deoxystrigol and strigol	Root length	Root diameter	Lateral root length	Lateral root density	Fine root number	Fine root density
1	*VvD27*	-0.967	0.557	0.956	0.441	0.379	0.862	0.918	0.991	0.606
2	*VvMAX2*	-0.181	0.786	0.364	0.862	0.894	0.441	-0.327	-0.211	-0.839
3	*VvCCD8*	-0.627	0.986	0.122	0.999*	0.999*	0.815	-0.737	0.279	-0.479
4	*VvCCD7*	-0.631	0.986	0.128	1*	0.999*	0.818	-0.741	0.284	-0.474
5	*VvMAX1*	0.948	0.921	0.638	0.861	0.824	0.999*	-0.985	0.753	0.07
6	*VvDAD2*	-0.772	0.158	0.991	0.025	-0.044	0.57	-0.667	0.957	0.883
7	*VvSMAX1*	-0.984	0.855	0.744	0.778	0.733	0.995	-1*	0.841	0.215
8	*VvSMAXL4*	0.887	0.36	-0.997*	0.232	0.165	0.729	-0.808	0.996	0.766
9	*VvSMAXL3a*	0.988	0.636	0.922	0.528	0.469	0.908	-0.953	0.972	0.524
10	*VvSMAXL3b*	0.993	0.824	0.781	0.741	0.693	0.988	-1*	0.87	0.27
11	*VvSMAXL6a*	0.656	-0.007	0.955	-0.14	-0.208	0.427	-0.535	0.896	0.949
12	*VvSMAXL6b*	-0.993	0.944	-0.587	0.892	0.859	0.994	-0.972	0.709	0.006

"*" after the correlation coefficients represent the correlation between their mRNA expression levels and endogenous hormone levels and root parameters significantly different at $P < 0.05$.
