# Supplementary material for: The Role of Strigolactones in the Regulation of Root System Architecture in Grapevine (Vitis vinifera L.) in Response to Root-Restriction Cultivation
Source: Int J Mol Sci. 2021 Aug 16;22(16):8799. doi: 10.3390/ijms22168799 (PMC8395845; doi:10.3390/ijms22168799)
Supplement: Supplementary file 1 [file ijms-22-08799-s001.zip › Table S6.pdf]

Table S6. Correlation between SLs-related gene expression levels and the parameters and SLs content of grapevine roots at 5 DAA.

Code	Annotation	(±)-2'-epi-5-deoxystrigol	strigol	(±)-2'-epi-5-deoxystrigol and strigol	Root length	Root diameter	Lateral root length	Lateral root density	Fine root number	Fine root density
1	*VvD27*	0.557	0.987	0.771	0.881	0.217	0.459	-0.298	0.108	-0.193
2	*VvMAX2*	0.786	0.932	0.339	0.999*	-0.31	0.847	-0.742	0.599	0.333
3	*VvCCD8*	0.986	-0.962	0.426	-0.852	0.22	-0.794	0.676	-0.522	-0.244
4	*VvCCD7*	0.986	0.976	0.56	-0.979	0.272	0.408	-0.243	0.052	-0.249
5	*VvMAX1*	0.921	0.848	0.03	-0.937	-0.481	0.931	-0.854	0.737	0.502
6	*VvDAD2*	0.158	0.634	1*	-0.2	0.807	-0.242	0.407	-0.575	-0.793
7	*VvSMAX1*	0.855	-0.24	0.889	0.876	-0.983	0.635	-0.76	0.871	0.978
8	*VvSMAXL4*	0.36	0.777	0.699	-0.399	0.672	-0.041	0.214	-0.398	-0.654
9	*VvSMAXL3a*	0.636	0.998*	0.436	-0.669	0.126	0.539	-0.384	0.199	-0.102
10	*VvSMAXL3b*	0.824	0.999*	0.173	-0.847	0.11	0.552	-0.399	0.214	-0.087
11	*VvSMAXL6a*	-0.007	-0.409	0.418	-0.035	0.888	-0.976	0.999*	-0.989	-0.899
12	*VvSMAXL6b*	0.944	0.999*	0.631	-0.957	0.022	0.624	-0.479	0.3	0.002

"*" after the correlation coefficients represent the correlation between their mRNA expression levels and endogenous hormone levels and root parameters significantly different at $P < 0.05$.
