# Supplementary material for: The Role of Strigolactones in the Regulation of Root System Architecture in Grapevine (Vitis vinifera L.) in Response to Root-Restriction Cultivation
Source: Int J Mol Sci. 2021 Aug 16;22(16):8799. doi: 10.3390/ijms22168799 (PMC8395845; doi:10.3390/ijms22168799)
Supplement: Supplementary file 1 [file ijms-22-08799-s001.zip › Table S7.pdf]

Table S7. Sequence of primers used for real-time PCR.

Gene name	Accession No.	Forward primer sequences (5’→3’)	Reverse primer sequences (5’→3’)
KyActin1	AB073011	GATTCTGGTGATGGTGTGAGT	GACAATTTCCCGTTCAGCAGT
VvMAX1	LOC100243924	CCCTCTCCACCAAAAGGGTC	GAATGCTTGCATGGTTGGCA
VvD27	LOC100853223	GCTGCCATGGTGTCAAGGAA	ATCACCTTGCAGGGTCCAAC
VvCCD8	LOC100250765	GAAGCCGGGGTACATAGTGG	GGGTGCCACTCGAACTTGTA
VvCCD7	LOC100242750	AAAATGTGGCGAACACCAGC	TCCGGCATCTTGAACACTCC
VvDAD2	LOC100257558	AAGGTGCCCTGCTGCATAAT	GGACCGGAGCCAGTAACATC
VvMAX2	LOC104880827	TCGGATTCAACGACTGCACA	GCTGGGGATACTTGGTTGCT
VvSMAX1	LOC100260369	TGGCTATTGTTCACGGGTCC	CTGCTTCTGCAATGCGATCC
VvSMAXL3a	LOC100254493	GTGTCTGGCGACTATCGAGG	CCTCTTCCCACGCAACTCTT
VvSMAXL3b	LOC100254987	CGAATGTGGCTTTTGGGGAC	CTTACTTTGGAACCGGCCCT
VvSMAXL4	LOC100247938	GGAGGAACACGACAAGCTCA	ATCATCCTTTTGGCGGGCTT
VvSMAXL6a	LOC100260392	GATTTGAACCTCCCGGCTGA	TGGTCTCGTGGAACGTCTTG
VvSMAXL6b	LOC100241532	AGACTACGTCCCTACACGCT	GGAGGATGGCAAACGATCCA
